# High quality assemblies of four indigenous chicken genomes and related functional data resources

**DOI:** 10.1038/s41597-024-03126-1

**Published:** 2024-03-15

**Authors:** Siwen Wu, Kun Wang, Tengfei Dou, Sisi Yuan, Shixiong Yan, Zhiqiang Xu, Yong Liu, Zonghui Jian, Jingying Zhao, Rouhan Zhao, Xiannian Zi, Dahai Gu, Lixian Liu, Qihua Li, Dong-Dong Wu, Junjing Jia, Zhengchang Su, Changrong Ge

**Affiliations:** 1 Faculty of Animal Science and Technology, Yunnan Agricultural University, Kunming, Yunnan 650201 China; 2 Department of Bioinformatics and Genomics, The University of North Carolina at Charlotte, Charlotte, NC 28223 USA; 3 State Key Laboratory of Genetic Resources and Evolution, Kunming Institute of Zoology, Chinese Academy of Sciences, Kunming, China; 4 Center for Excellence in Animal Evolution and Genetics, Chinese Academy of Sciences, Kunming, China

**Keywords:** Genome, Genome assembly algorithms

## Abstract

Many lines of evidence indicate that red jungle fowl (RJF) is the primary ancestor of domestic chickens. Although multiple versions of RJF (galgal2-galgal5 and GRCg6a) and commercial chickens (GRCg7b/w and Huxu) genomes have been assembled since 2004, no high-quality indigenous chicken genomes have been assembled, hampering the understanding of chicken domestication and evolution. To fill the gap, we sequenced the genomes of four indigenous chickens with distinct morphological traits in southwest China, using a combination of short, long and Hi-C reads. We assembled each genome (~1.0 Gb) into 42 chromosomes with chromosome N50 90.5–90.9 Mb, amongst the highest quality of chicken genome assemblies. To provide resources for gene annotation and functional analysis, we also sequenced transcriptomes of 10 tissues for each of the four chickens. Moreover, we corrected many mis-assemblies and assembled missing micro-chromosomes 29 and 34–39 for GRCg6a. Our assemblies, sequencing data and the correction of GRCg6a can be valuable resources for studying chicken domestication and evolution.

## Background & Summary

Evidence shows that red jungle fowl (RJF) (*Gallus gallus*) is the primary ancestor of domestic chickens (*Gallus gallus domesticus*) all over the world^1^. Since the release of the initial draft genome assembly (galgal2) of an RJF individual^2^, multiple improved assemblies (galgal3-galgal5 and GRCg6a) have been developed^3,4^. More recently, the Vertebrate Genomes Project (VGP) also assembled pseudo-haplotype genomes (GRCg7b and GRCg7w) of a hybrid individual from a broiler mother and a layer father using long sequencing reads and multiple scaffolding data^5,6^. There are also several assemblies for indigenous chickens deposited in GenBank, such as Yeonsan Ogye chicken (Ogye1.0) and Tibetan chicken (ASM2537063v1). However, with a contig N50 < 1 M and lack of chr29 and chr34–39, their quality is quite low. Li, *et al*. have assembled a so-called pan-genome for 20 chicken breeds using short, long and Hi-C reads^7^. However, with a contig N50 of 5.89–16.72 Mb and a complete BUSCO^8^ value of 92.4%-95.3%, this assembly does not allow the identification of subtle differences in gene compositions of chickens. Besides, they failed to assemble many micro-chromosomes such as 29 and 34–39. Recently, gap-less genome assemblies for a Chinese broiler (Huxu chicken)^9^ and a Silkie chicken^10^ have been made using PacBio HiFi, Oxford Nanopore long reads and multiple scaffolding data. In another effort, Rice, *et al*. generated a pangenome graph based on 30 assembled chicken genomes, including the GRCg7b/w and Huxu assemblies, among others^11^. Although these assemblies provide a good foundation for understanding various aspects of chicken biology and guiding poultry breeding, more high-quality assemblies for indigenous chickens (traditionally domesticated village chickens) are necessary to further the understanding of chicken domestication and evolution.

To fill the gaps, we sequenced and assembled genomes of four individual indigenous chickens of Daweishan, Hu, Piao and Wuding breeds from Yunnan province in southwestern China, one of the major geographical places where the domesticated chickens originated^1^. These chicken breeds have been developed by less-intensive traditional family-based artificial selection in villages in isolated mountainous areas in the province since 2000–6000 BC^12^. Each breed possesses distinct morphological traits: Daweishan chickens have a miniature body size (0.5–0.8 kg for female and 0.8–1.2 kg for male adults); Hu chickens have a large body size (3 kg for female and 6 kg for male adults) with extraordinarily stout legs; Piao chickens have a short tail (a rumpless phenotype); and Wuding chickens have a middle-sized body and are good at running. Using a combination of Illumina short, PacBio or Oxford Nanopore long and Hi-C reads, we assembled each genome at the chromosome-level with a contig N50 of 16.2–25.1 Mb, chromosome N50 of 90.5–90.9 Mb and a complete BUSCO value of 96.5%-96.7%. Evaluations from multiple aspects of criteria proposed by the Vertebrate Genome Project^5^ suggest that our assemblies are amongst the highest quality of chicken genome assemblies. To provide resources for gene annotation and functional analysis of indigenous chickens, we also sequenced the transcriptomes of 10 tissues for each of the four indigenous chickens. In addition, we corrected many mis-assemblies in micro-chromosomes (31–33) and assembled micro-chromosomes 29 and 34–39 for RJF (GRCg6a), providing a more complete assembly for the primary ancestor of domestic chickens. Our assembled high-quality indigenous chicken genomes, related functional data, and corrections of the GRCg6a assembly can be valuable resources for the community to reveal the genetic basis of the important and interesting traits of these chicken breeds as well as to study the evolution and domestication process of chickens.

## Methods

### Chickens

A female individual of Daweishan chicken aged 10 months, a female individual of Hu chicken aged 7 months, a female individual of Piao chicken aged 10 months and a female individual of Wuding chicken aged 10 months were collected from corresponding chicken breed populations from the Experimental Breeding Chicken Farm of the Yunnan Agricultural University (Yunnan, China). Each individual chicken was subject to Illumina short-reads DNA sequencing, PacBio or Oxford Nanopore long-reads sequencing, Hi-C sequencing and Illumina short-reads RNA sequencing.

### Ethics statement

All the experimental procedures were approved by the Animal Care and Use Committee of the Yunnan Agricultural University (approval ID: YAU202103047). The care and use of animals fully complied with local animal welfare laws, guidelines, and policies.

### Short-reads DNA sequencing

Two milliliters of blood were drawn from the wing vein of each chicken in a centrifuge tube containing anticoagulant (EDTA-2K) and stored at −80 °C until use. Genomic DNA (10 µg) in each blood sample was extracted using a DNA extraction kit (DP326, TIANGEN Biotech, Beijing, China) and fragmented using a Bioruptor Pico System (Diagenode, Belgium). DNA fragments around 350 bp were selected using SPRI beads (Beckman Coulter, IN, USA). DNA-sequencing libraries were prepared using Illumina TruSeq® DNA Library Prep Kits (Illumina, CA, USA) following the vendor’s instructions. The libraries were subject to 150 cycles paired-end sequencing on an Illumina Novaseq 6000 platform (Illumina, CA, USA) at 100X coverage.

### PacBio long-reads sequencing

Two milliliters of blood were drawn from the wing vein of a female Hu chicken (H3) in a centrifuge tube with anticoagulant (EDTA-2K) and stored at −80 °C until use. High molecular weight DNA was extracted from each blood sample using NANOBIND® DNA Extraction Kits (PacBio, CA, USA) following the vendor’s instructions. DNA fragments of about 25 kb were size-selected using a BluePippin system (Sage Science, MA, USA). Sequencing libraries were prepared for the DNA fragments using SMRTbell® prep kits (PacBio, CA, USA) following the vendor’s instructions, and subsequently sequenced on a PacBio Sequel II platform (PacBio, CA, USA) at 36X coverage.

### Oxford Nanopore long-reads sequencing

Two milliliters of blood were drawn from the wing vein of a female Daweishan (F025), Piao (P17) or Wuding (W17) chicken in a centrifuge tube with anticoagulant (EDTA-2K) and stored at −80°C until use. High molecular weight DNA from each blood sample was prepared using Ultra-Long Sequencing Kits (Oxford Nanopore Technology (ONT), Oxford, UK) following the vendor’s instructions. The integrity of DNA was determined using pulsed field electrophoresis. DNA fragments of about 20 kb were size-selected using a BluePippin system (Sage Science, MA, USA). Sequencing libraries were prepared for the DNA fragments using ONT Template prep kit (SQK-LSK109) and NEB Next FFPE DNA Repair Mix kit, following the vendors’ instructions. The libraries were sequenced on a Nanopore PromethION P48 platform (ONT, Oxford, UK) at ~100X coverage using ONT sequencing kits (EXP-FLP001.PRO.6).

### Hi-C sequencing

Five milliliter of blood were drawn from the wing vein of the selected Daweishan (F025), Piao (P17), Hu chicken (H3) or Wuding (W17) chickens in a Streck Cell-free DNA BCT collecting vessel (Streck Corporate, USA), and stored at 4 °C and used in 24 hours. Hi-C libraries were constructed using Phase Genomics’ Animal Hi-C kit following the vendor’s instructions and subsequently sequenced on an Illumina’s Novaseq 6000 platform at a sequencing depth of ~100X.

### Transcriptome sequencing

One to two grams of various tissues were collected from the selected female individual chicken of each breed in a centrifuge tube and immediately frozen in liquid nitrogen, then stored at −80 °C until use. Total RNA from each tissue sample were extracted using TRlzol reagents (TIANGEN Biotech, Beijing China) according to the manufacturer’s instructions. RNA-sequencing libraries for each tissue as well as for the mixture of all the tissues collected from a chicken were prepared using Illumina TruSeq® RNA Library Prep Kits (Illumina, San Diego) following the vendor’s instructions. The libraries were subject to 150 cycles paired-end sequencing on an Illumina Novaseq 6000 platform at a sequencing depth of 80X. Additional individual chicken was randomly selected from each breed population and the same types of tissues were collected. Total RNA from each tissue sample were extracted in the same way as described above. Equal weight of total RNA of each tissue was mixed for preparing an RNA-seq library as described above.

### Quality assessment of sequencing data

We used FastQC (0.12.1) (http://www.bioinformatics.babraham.ac.uk/projects/fastqc) to evaluate the quality of our different kinds of sequencing data. Default parameters were used in the tool.

### Contig assembling

We filtered out PacBio/Oxford Nanopore long reads shorter than 5,000 bp in each library, and assembled contigs using Wtdbg (2.5)^13^ with the remaining reads for each chicken. Parameters used in the tool for each chicken are summarized in Table S1.

### Scaffolding and gap filling

We bridged the contigs by 100 Ns and obtained scaffolds using SALSA^14,15^ with the Hi-C sequencing reads. We filled the gaps in the scaffolds using PBJelly^16^ with PacBio/Oxford Nanopore long reads, and made two rounds of polishing on the resulting scaffolds for each chicken, first by using Racon (1.4.21)^17^ with the long reads, and second by using NextPolish (1.4.0)^18^ with the paired-end short reads from the same individual chicken. Parameters used in each tool for each chicken are summarized in Table S1.

### Chromosome-level genome assembling

To sort the assembled scaffolds into chromosomes, we used the chromosomes of the GRCg7b assembly as templates, except for chr16 where we used that in the Huxu chicken assembly (GGswu), since this chromosome is much more completely assembled in the Huxu reference than in GRCg7b (4.9 Mb vs 2.7 Mb). Specifically, we mapped the assembled scaffolds in each chicken to the templates using blastn (2.11.0)^19^. The parameters used in the tool for each chicken are summarized in Table S1. We consider a scaffold as being mapped to a chromosome if the ratio of the mapped length of a scaffold over the minimum of the length of the query scaffold and the length of the target chromosome was greater than 0.5. We ordered and orientated the scaffolds based on their mappings on a template chromosome. We concatenated the scaffolds mapped to the same chromosome by 500 Ns according to their mapping orders. The remaining scaffolds that could not be mapped to the template chromosomes, were considered as unplaced scaffolds. In this way, we sorted the assembled scaffolds of the four chickens into 42 chromosomes (including two sex chromosomes and one mitochondrial chromosome).

During the mapping, we found that, in two cases, one part of a scaffold was mapped to one template chromosome while the other part mapped to another chromosome. Each section was connected by 100 Ns, suggesting that two parts of two different chromosomes were incorrectly concatenated into a scaffold by the scaffolding tool, probably due to the physical proximity of the territories of the two chromosomes in the nuclei. We thus manually split the two parts and sorted them into their corresponding mapped template chromosomes. In addition, there is no scaffold in Hu chicken’s primary assembly, which can be mapped to the mitochondrial chromosome of GRCg7b. Thus, it appears that the assembly tools did not assemble the mitochondrial chromosome for Hu chicken for some unknown reason. We therefore mapped the short reads of Hu chicken to the mitochondrial chromosome of GRCg7b, and then assembled the mitochondrial chromosome for Hu chicken using Abyss (2.2.5)^20^ with default settings using the mapped short reads.

### Quality evaluation of assemblies

We masked the repeats of the four assemblies using WindowMasker (2.11.0)^21^, and estimated the heterozygosity of each assembly using Jellyfish (2.3.0)^22^ and GenomeScope^23^. To estimate the continuity of each assembly, we used QUAST (5.0.2)^24^ to calculate the contig N50, scaffold N50 and chromosome N50. To estimate the structural accuracy, we used Asset (https://github.com/dfguan/asset) to calculate the reliable block N50 and used BUSCO (5.1.3)^8^ to calculate the false duplications in each assembly. To estimate the base accuracy, we used Merqury (1.3)^25^ to calculate the k-mer QV and k-mer completeness for the four assemblies (k = 17), used BWA (0.7.17)^26^ to map the short reads to the four assemblies, and used and SAMtools (1.10)^27^ to analyze the mapping results. To estimate the functional completeness, we used BUSCO (5.1.3)^8^ to assess each assemblies’ completeness against the avian gene set and used STAR (2.7.0)^28^ to map the mRNA short reads to each assembly and calculate the mRNA completeness value. To plot the heatmap of the chromosomes of each assembly, we mapped the Hi-C paired-end reads to the assembly using BWA (0.7.17)^26^, used SAMtools (1.10)^27^ and Pairtools (0.3.0) (https://github.com/open2c/pairtools) to analyze the mapping results, and used Higlass^29^ to plot the heatmap for each assembly. Default parameters were used in each tool.

### Correction of GRCg6a assembly

Since chromosomes 29 and 34–39 of the most recent RJF genome assembly (GRCg6a) are still missing and some assembled chromosomes might contain mis-assemblies, we assembled these missing chromosomes to provide a better reference assembly for RJF. We validated these newly assembled chromosomes using the GRCg7b reference as the template, except chr16 for which we used that in the Huxu chicken assembly (GGswu) for the aforementioned reason. Specifically, we mapped all the assembled chromosomes and unplaced contigs of GRCg6a to the templates using blastn (2.11.0)^19^ with the same parameters used in the four indigenous chickens. If a chromosome of GRCg6a was completely mapped to the corresponding template chromosome, then we kept it intact. If the contigs of a chromosome from GRCg6a were mapped to different template chromosomes (indicating possible mis-scaffolding), we split them between the Ns that concatenated them, and sorted the split contigs to the mapped template chromosomes. Finally, we assembled the missing chromosomes and mis-assembled ones by ordering, orientating and concatenating (by 500 Ns) the split and unplaced contigs based on their mappings on the template chromosomes.

## Data Records

All the Illumina short DNA sequencing reads, PacBio or Oxford Nanopore long reads, Hi-C reads and the Illumina short RNA-seq reads of different tissues of the four indigenous chickens are available at NCBI SRA database with accession number PRJNA865263^30^. The assembled genomes of Daweishan, Hu, Piao and Wuding chicken have passed NCBI’s quality evaluation and are available at NCBI GenBank under the BioProject number PRJNA865263^31–34^. Details of each sample in the BioProject are summarized in Table S2. The corrected and assembled missing chromosomes of GRCg6a are available at the figshare database^35^.

## Technical Validation

### Quality evaluation of the sequencing data of the four chicken breeds

To assemble the genome of an individual of each of the four indigenous chicken breeds, we generated Illumina paired-end short reads, PacBio or Oxford Nanopore long reads and Hi-C paired-end short reads for each selected individual. As shown in Table 1, the Illumina paired-end DNA short reads have a length 150 bp and cover from 89X in Hu chicken to 143X in Daweishan chicken genomes. The long reads in Hu chicken were generated using PacBio sequencing with an average length 12.6 kbp, covering 36X of the genome. The long reads in the other three chickens were produced using Oxford Nanopore sequencing with an average length ~12 kbp, covering about 100X of the genomes. For the Hi-C paired-end short reads, the sequencing length is 150 bp with about 100X sequencing depth for all the four chickens. To provide resources for gene annotation, we also sequenced transcriptomes of 10 tissues in each of the four chickens using paired-end RNA-seq with a sequencing length 150 bp and 257–280 million reads.

**Table 1:** Summary of sequencing data from the four chicken breeds.

		Daweishan	Hu	Piao	Wuding
**Short reads**	**Depth**	143	89	116	132
	**Length**	150 bp	150 bp	150 bp	150 bp
	**# Pairs**	49,77,66,684	29,99,89,652	40,46,41,183	46,15,06,229
**Long reads**	**Type**	Nanopore	PacBio	Nanopore	Nanopore
	**Depth**	109	36	110	101
	**Average reads length**	14.5kbp	12.6kbp	10.1kbp	12.0kbp
	**# Total reads**	78,05,570	29,02,816	1,13,51,310	88,09,621
	**# Reads > 5kbp**	49,67,248	20,86,941	73,08,102	51,35,718
**Hi-C reads**	**Depth**	108	112	102	110
	**Length**	150 bp	150 bp	150 bp	150 bp
	**# Pairs**	37,42,69,265	37,64,95,854	35,44,82,162	38,43,54,246
**RNA-seq reads**	**Length**	150 bp	150 bp	150 bp	150 bp
	**# Tissues**	10	9	10	10
	**# Pairs**	25,73,50,330	26,64,74,857	28,02,08,923	26,98,18,596

**Fig. 1:**
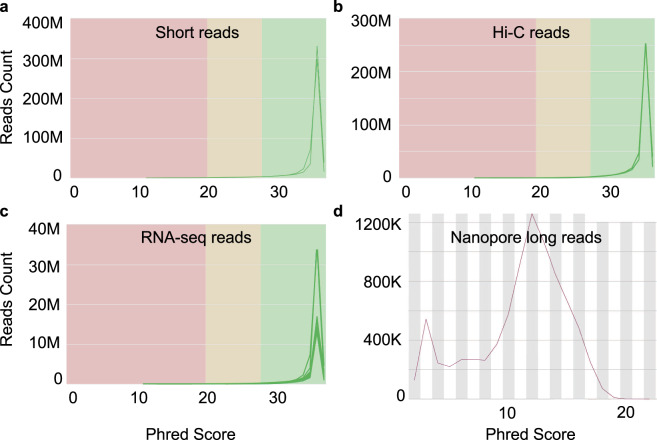
Quality assessment of different types of sequencing reads for the Wuding chicken individual.

Figure 1 shows the results of quality assessment of these different kinds of sequencing data of Wuding chicken. All the Illumina reads including genomic paired-end short reads, Hi-C paired-end short reads and RNA-seq paired-end short reads of the Wuding chicken individual have a phred quality score greater than 35, suggesting that the base accuracy of all these reads is greater than 99.9% (https://www.illumina.com/documents/products/technotes/technote_Q-Scores.pdf). For the Oxford Nanopore long reads of the Wuding chicken individual, the peak phred quality is about 12. Similar results were obtained for the sequencing data of the other three chickens (Supplementary Figures). Of note, PacBio sequencing reads do not come with phred quality scores, thus, the long reads of Hu chicken were evaluated using length distribution, which indicates the data is of high quality.

### Evaluation of the quality of the assemblies of the four indigenous chicken genomes

Using a combination of Illumina short reads (89–143X) and PacBio (36X) or Oxford Nanopore (101-110X) long reads (Table 1), we assembled the genomes of a female individual of the Daweishan, Hu, Piao and Wuding chicken into 462–1,364 contigs (Table S3) with a contig N50 of 23.0, 16.2, 25.1 and 21.5 Mb, respectively. Hu chickens’ smaller contig N50 (16.2 Mb) might be due to the shorter PacBio reads (50 bp-90.8 kbp) and a shallower sequencing depth (36X) than those of Oxford Nanopore reads (Table 1). The contig N50 values of the Daweishan, Piao and Wuding assemblies are larger than those of the GRCg6a, GRCg7b and GRCg7w assemblies (17.7–18.8 Mb) (Table 2). The total length of the contigs (>1 Gb) for each chicken is comparable with those of GRCg6a, GRCg7b and GRCg7w assemblies (Table 2). We scaffolded the contigs using Hi-C reads (102-112X) for each chicken (Table 1), resulting in 308-1,088 scaffolds, with a scaffold N50 of 74.3, 28.9, 62.8 and 71.1 Mb for the Daweishan, Hu, Piao and Wuding chickens, respectively (Table 2). Using the GRCg7b chromosomes and chr16 of the Huxu chicken assembly (GGswu) as the template, we ordered and oriented the scaffolds into 39 autosomal chromosomes, two sex chromosomes W and Z and one mitochondrial genome for each chicken (Table S3), with a chromosome N50 of 90.5, 90.7, 90.5 and 90.9 Mb for the Daweishan, Hu, Piao and Wuding chickens, respectively, comparable to those of the GRCg6a and GRCg7b/w assemblies (Table 2).

**Table 2:** Evaluation of the four assemblies using six categories of criteria.

Chicken	Genome		Continuity					Structural Accuracy		Base Accuracy			Functional Completeness		Chromosome Status		
	Heterozygosity (%)	Repeats (%)	Size (Gb)	Contig N50 (Mb)	Scaffold N50 (Mb)	Chromosome N50 (Mb)	# Gaps	Reliable block N50 (Mb)	False duplications (%)	*k-mer* QV	*k-mer* Completeness (%)	Short reads Completeness (%)	BUSCO Completeness (%)	mRNA Completeness (%)	Assigned to Chromosome (%)	Mitochondria (kbp)	Sex Chromosome
**Daweishan**	0.8	20.3	1.037	23.0	74.3	90.5	388	17.1	0.3	41.6	92.9	99.4	96.7	95.0	98.8	16.0	ZW
**Hu**	0.7	20.0	1.027	16.2	28.9	90.7	515	2.9	0.3	38.4	93.3	99.3	96.6	91.3	98.9	16.5	ZW
**Piao**	0.9	20.4	1.036	25.1	62.8	90.5	506	13.7	0.3	41.6	92.9	99.5	96.6	95.0	98.3	16.3	ZW
**Wuding**	0.8	19.9	1.026	21.5	71.1	90.9	312	18.7	0.4	43.3	93.1	99.3	96.5	95.2	99.2	16.8	ZW
**RJF(GRCg6a)**	—	20.4	1.056	17.7	—	91.3	5,00,945	—	0.4	—	—	—	96.6	—	98.6	16.8	ZW
**Broiler(GRCg7b)**	—	20.5	1.050	18.8	—	90.9	463	—	0.4	—	—	—	96.6	—	98.8	16.8	ZW
**Layer(GRCg7w)**	—	20.2	1.046	17.7	—	90.6	409	—	0.4	—	—	—	96.8	—	98.5	—	ZW

Recently, the VGP consortium proposed six categories of criteria for evaluating the quality of a chromosome-level assembly, including genome (degree of heterozygosity and repeats), continuity, structural accuracy, base accuracy, functional completeness, and chromosomal assignment status^5^. We thus further evaluated the quality of each of our assembled genomes using these criteria (Table 2). For the genome evaluation, we found that Piao chicken had the highest heterozygosity of 0.9%, Hu chicken the lowest heterozygosity of 0.7%, and both Daweishan and Wuding chicken a middle heterozygosity of 0.8%. Repeats consist of from 19.9 to 20.4% of all the four assembled genomes, which are similar to those of GRCg6a (20.4%), GRCg7b (20.5%) and GRCg7w (20.2%). For the continuity evaluation, both the contig N50 (16.2–25.1 Mb) and chromosome N50 (90.5–90.9 Mb) of our assemblies are comparable with those of GRCg6a (17.7 and 91.3 Mb), GRCg7b (18.8 and 90.9 Mb) and GRCg7w (17.7 and 90.6 Mb). There are 388, 515, 506 and 312 gaps in the Daweishan, Hu, Piao and Wuding assemblies, respectively, which are substantially fewer than those in GRCg6a (500,945) and are comparable with those in GRCg7b (463) and GRCg7w (409). For the structural accuracy evaluation, we identified reliable blocks and false duplications of the assemblies. As shown in Table 2, we achieved a reliable block N50 > 13.5 Mb except for Hu chicken (2.9 Mb), and a false duplication rate of 0.3–0.4%. The values of both parameters are comparable to those of the recent VGP assemblies of 16 species of six major vertebrate lineages^5^.

For the base accuracy evaluation, we first computed the k-mer QVs of our assemblies, which is the log-scaled probability of consensus errors in the assembly^25^. We found that the k-mer QVs of the Daweishan, Piao and Wuding chicken assemblies were greater than 41.5 and the value of the Hu chicken assembly was 38.4, suggesting that the consensus base accuracy is greater than 99.99% and 99.90% for the former three assemblies and the Hu chicken assembly^25^, respectively, which is comparable to those obtained by the VGP assemblies^5^. We next calculated k-mer completeness, which is defined as the fraction of reliable k-mers in highly accurate short reads data that are also found in the assembly^25^. As shown in Table 2, the k-mer completeness for all the four assemblies is greater than 92.8%, also comparable to those of the recent VGP assemblies^5^. Since our assemblies are the mosaics of the paternal and maternal homologous chromosomes that differ in heterozygous sites, to further evaluate the completeness of the assemblies, we mapped short reads from each individual chicken to its assembled genome and found that the mapping rates were greater than 99.2% for all the assemblies. These results indicate that all our four assemblies have achieved high base accuracy.

For the functional completeness evaluation, all of the four assemblies have >96.5% BUSCO completeness^8^, which are comparable to those of GRCg6a (96.6%), GRCg7b (96.6%) and GRCg7w (96.8%). We next mapped the RNA-seq reads from multiple tissues of each chicken to its assembled genome and found that the mapping rates were at least 95.0% for all the four assembled genomes except for Hu chicken (91.3%). These results indicate that the assemblies are of high completeness. For the chromosomal assignment status evaluation, although there are still some unplaced contigs in each of our assemblies, the total length (non-N bp) of the contigs assigned to chromosomes are greater than 98% for all the four assemblies, which are similar to those of GRCg6a and GRCg7b/w. Thus, most of our contigs are assigned to chromosomes.

**Fig. 2:**
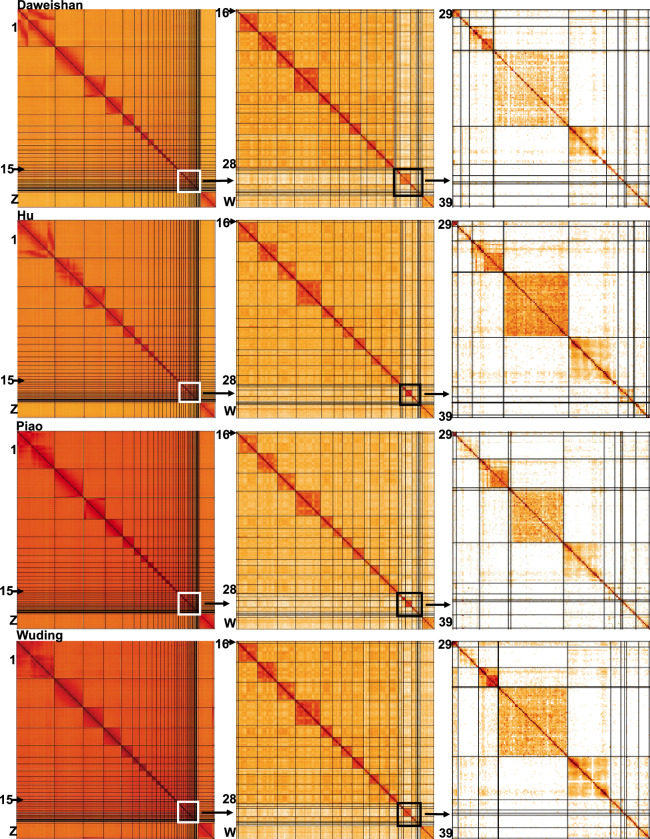
Interaction heatmaps of the assembled autosomal and sex chromosomes of the four chicken genomes. First column: heatmaps of all the 41 chromosomes of Daweishan, Hu, Piao and Wuding chicken. Second column: a zoomed-in view of the heatmaps of chr16-chr28 and sex chromosome W of Daweishan, Hu, Piao and Wuding chicken. Third column: a zoomed-in view of the heatmaps of chr29-chr39 of Daweishan, Hu, Piao and Wuding chicken.

**Fig. 3:**
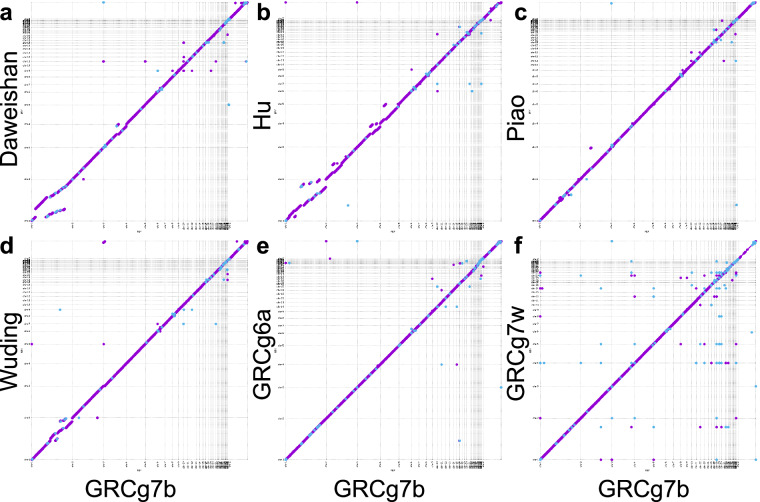
Dot plots showing collinearity of assembled chromosomes of the four indigenous chickens, RJF and GRCg7w with respect to corresponding chromosomes in GRCg7b.

Additionally, we plotted the Hi-C interaction heatmaps of the autosomes and sex chromosomes (W and Z) of each of the four assemblies. As shown in Fig. 2, for all the four genomes, most assembled chromosomes including the micro-chromosomes (chr11-39) form a squared box along the main diagonal of the heatmap matrix, with the exception of a few very small chromosomes such as chr31 and chr35. Moreover, the assembled chromosomes in each of the four assembled genomes display high collinearity with those of the GRCg7b reference (Fig. 3), indicating that the structures of the assembled genomes are consistent. Moreover, our assembled mitochondrial genomes of Daweishan, Hu, Piao and Wuding chickens have a length of 16.0, 16.5, 16.3 and 16.8 kbp, respectively, which are similar to those of GRCg6a (16.8 kbp), GRCg7b (16.8 kbp) and GRCg7w (16.8 kbp) (Table 2). Taken together, these results indicate that we have achieved chromosome-level assembly for all four chicken genomes with very high quality.

### Assembly of missing micro-chromosomes and correction of mis-assemblies in GRCg6a

The current RJF’s reference genome (GRCg6a) lacks assemblies of micro-chromosomes 29 and 34–39. We found that contigs of these chromosomes were either mistakenly assembled into chr31, chr32 and chr33 or were unplaced (Methods). We assembled these missing chromosomes and corrected the mis-assembled chr31, chr32 and chr33 of GRCg6a using the corresponding chromosomes in the GRCg7b assembly as templates (Methods). Table 3 summarizes the contig compositions and lengths of these assembled RJF micro-chromosomes in comparison with those of the GRCg7b assembly. Interestingly, except for chr35, chr36 and chr39, these assembled RJF micro-chromosomes are much longer than their corresponding ones in GRCg7b, suggesting that they might be more complete. Chr1-28, chr30, the two sex chromosomes and the mitochondrial chromosome in GRCg6a were consistent with those of GRCg7b, and thus were kept intact. The assembled GRCg6a chromosomes also display high collinearity with those of GRCg7b (Fig. 3), indicating that the structures of the two assembled genomes are consistent.

**Table 3:** Contig compositions and lengths of the assembled RJF chr29, chr31-chr39 in comparison with the corresponding chromosomes of the GRCg7b assembly.

Chromosomes	Length in GRCg7b (non-N bp)	Number of contigs in GRCg6a				Length in GRCg6a (non-N bp)	Percentage
		Chr31	Chr32	Chr33	Unplaced contigs		
**Chr29**	5,54,358	17	—	2	4	19,36,443	349.31%
**Chr31**	24,54,334	27	—	4	61	48,22,944	196.51%
**Chr32**	1,25,424	—	—	—	6	6,94,328	553.58%
**Chr33**	26,79,370	1	—	3	6	29,26,464	109.22%
**Chr34**	33,88,267	9	—	22	93	57,71,600	170.34%
**Chr35**	5,53,226	1	—	—	7	3,44,272	62.23%
**Chr36**	3,57,675	—	—	3	7	1,50,317	42.03%
**Chr37**	1,57,653	—	—	1	6	4,57,626	290.27%
**Chr38**	6,66,612	—	6	—	—	7,25,311	108.81%
**Chr39**	1,77,356	—	—	1	—	1,67,109	94.22%

### Varying lengths and high G/C contents of assembled micro-chromosomes chr16 and chr29-39

**Fig. 4:**
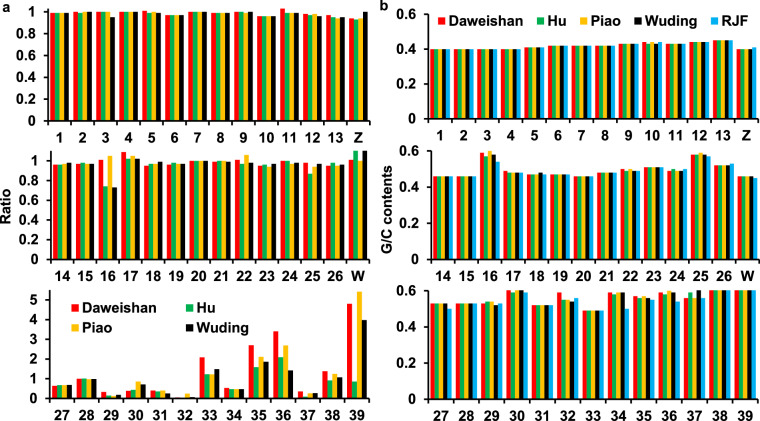
Comparison of the length and G/C contents of each chromosome of the five chickens. **a**. Ratio of the length of each chromosome (excluding Ns) of the four indigenous chickens over that of the same chromosome (excluding Ns) of RJF. **b**. G/C contents of each chromosome of the five chickens.

We compared the lengths of the assembled chromosomes of the four indigenous chickens with those of RJF. As shown in Fig. 4a,the four indigenous chickens and RJF have similar lengths of all the assembled chromosomes, except for micro-chromosomes 16 and 29–39 that show highly varying lengths. These results indicate that the lengths of all the assembled macro-chromosomes (chr1-10), most micro-chromosomes (chr11-15, 17–26) and sex chromosome (chrW and chrZ) of the five chickens are consistent, and thus are sufficiently assembled. Our assemblies of chr33, chr35, chr36, chr38 and chr39 are much longer than those of RJF, but our assemblies of chr27, chr29, chr31, chr32, chr34 and chr37 are shorter than those of RJF. These micro-chromosomes have higher G/C contents than macro-chromosomes (Fig. 4b). Thus, the difficulty to better assemble these micro-chromosomes might be at least partially due to their higher G/C contents, since genome regions with high G/C content are difficult to sequence using the technologies we used to generate the DNA reads. The lengths of the assembled chr16 in Daweishan and Piao chickens are comparable to that of RJF; however, those in Hu and Wuding chickens are only 73% that of RJF (Fig. 4a,Table S3), even though we used chr16 (4.9 Mb) in Huxu chicken as the template to assemble the same chromosome in the four indigenous chickens (Methods). When a shorter chr16 (2.7 Mb) in GRCg7b was used as the template, the resulting chr16 assemblies in all the four indigenous chickens only had lengths <60% that of RJF (data not shown). Thus, in absence of accurate longer reads, chromosomes can be more accurately assembled by using more complete chromosomes as the templates. Interestingly, chr16 in Huxu chicken (4.9 Mb) is much longer than that in Silkie chicken (3.3 Mb), although chromosomes of both chickens were assembled using PacBio HiFi long reads, suggesting that different chicken breeds might have a variable chr16 that harbors highly varying repeat regions in the major histocompatibility complex (MHC)^36^. Thus, the variable lengths of the assembled chr16 in the different chicken breeds might be due to a combination of factors including their highly varying repetitive major MHC regions, relatively more duplicated genes^36^, and higher G/C contents (Fig. 4b). More efforts are needed in the future to completely assemble the micro-chromosomes in the indigenous chickens using new sequencing technologies.

### Supplementary information

Table 1

Table 2

## Data Availability

The genome assembly pipeline, codes and the documentation are available at https://github.com/zhengchangsulab/A-genome-assebmly-and-annotation-pipeline.
