# Author Correction: High quality assemblies of four indigenous chicken genomes and related functional data resources

**DOI:** 10.1038/s41597-024-03203-5

**Published:** 2024-04-08

**Authors:** Siwen Wu, Kun Wang, Tengfei Dou, Sisi Yuan, Shixiong Yan, Zhiqiang Xu, Yong Liu, Zonghui Jian, Jingying Zhao, Rouhan Zhao, Xiannian Zi, Dahai Gu, Lixian Liu, Qihua Li, Dong-Dong Wu, Junjing Jia, Zhengchang Su, Changrong Ge

**Affiliations:** 1 Faculty of Animal Science and Technology, Yunnan Agricultural University, Kunming, Yunnan 650201 China; 2 Department of Bioinformatics and Genomics, The University of North Carolina at Charlotte, Charlotte, NC 28223 USA; 3 State Key Laboratory of Genetic Resources and Evolution, Kunming Institute of Zoology, Chinese Academy of Sciences, Kunming, China; 4 Center for Excellence in Animal Evolution and Genetics, Chinese Academy of Sciences, Kunming, China

**Keywords:** Genome, Genome assembly algorithms, Genome, Genome assembly algorithms

Correction to: *Scientific Data* 10.1038/s41597-024-03126-1, published online 15 March 2024

In this article, affiliation 1 and 2 were inadvertently swapped, and the affiliation “Faculty of Animal Science and Technology, Yunnan Agricultural University, Kunming, Yunnan 650201, China” was missing for Siwun Wu. This original article has been corrected.

