# Supplementary material for: High quality assemblies of four indigenous chicken genomes and related functional data resources
Source: Sci Data. 2024 Mar 15;11:300. doi: 10.1038/s41597-024-03126-1 (PMC10942973; doi:10.1038/s41597-024-03126-1)
Supplement: Supplementary file 1 — Table 1 [file 41597_2024_3126_MOESM1_ESM.docx]

**Supplementary Figures**

Supplementary Figure 1-------------------------------------------------------------------------------------Page 2

Supplementary Figure 2-------------------------------------------------------------------------------------Page 3

Supplementary Figure 3-------------------------------------------------------------------------------------Page 4

**Supplementary Figure 1.** Quality assessment of different types of sequencing reads for the Daweishan chicken individual

**Supplementary Figure 2.** Quality assessment of different types of sequencing reads for the Hu chicken individual

**Supplementary Figure 3.** Quality assessment of different types of sequencing reads for the Piao chicken individual
